# Predictors for Delayed Emergency Department Care in Medical Patients with Acute Infections – An International Prospective Observational Study

**DOI:** 10.1371/journal.pone.0155363

**Published:** 2016-05-12

**Authors:** Alexander Kutz, Jonas Florin, Pierre Hausfater, Devendra Amin, Adina Amin, Sebastian Haubitz, Antoinette Conca, Barbara Reutlinger, Pauline Canavaggio, Gabrielle Sauvin, Maguy Bernard, Andreas Huber, Beat Mueller, Philipp Schuetz, for the TRIAGE Study group

**Affiliations:** 1 Division of General and Emergency Medicine, University Department of Medicine, Kantonsspital Aarau, Aarau, Switzerland; 2 Emergency department, Groupe Hospitalier Pitié-Salpêtrière, Assistance Publique-Hôpitaux de Paris (APHP), Paris, France; 3 Sorbonne Universités UPMC-Univ Paris06, UMRS INSERM 1166, IHUC ICAN, Paris, France; 4 Morton Plant Hospital, Clearwater, FL, United States of America; 5 Department of Clinical Nursing Science, Kantonsspital Aarau, Aarau, Switzerland; 6 Biochemistry Department, Hôpital Pitié-Salpêtrière and Univ-Paris Descartes, Paris, France; 7 Department of Laboratory Medicine, Kantonsspital Aarau, Aarau, Switzerland; University of Granada—Q1818002F, SPAIN

## Abstract

**Introduction:**

In overcrowded emergency department (ED) care, short time to start effective antibiotic treatment has been evidenced to improve infection-related clinical outcomes. Our objective was to study factors associated with delays in initial ED care within an international prospective medical ED patient population presenting with acute infections.

**Methods:**

We report data from an international prospective observational cohort study including patients with a main diagnosis of infection from three tertiary care hospitals in Switzerland, France and the United States (US). We studied predictors for delays in starting antibiotic treatment by using multivariate regression analyses.

**Results:**

Overall, 544 medical ED patients with a main diagnosis of acute infection and antibiotic treatment were included, mainly pneumonia (n = 218; 40.1%), urinary tract (n = 141; 25.9%), and gastrointestinal infections (n = 58; 10.7%). The overall median time to start antibiotic therapy was 214 minutes (95% CI: 199, 228), with a median length of ED stay (ED LOS) of 322 minutes (95% CI: 308, 335). We found large variations of time to start antibiotic treatment depending on hospital centre and type of infection. The diagnosis of a gastrointestinal infection was the most significant predictor for delay in antibiotic treatment (+119 minutes compared to patients with pneumonia; 95% CI: 58, 181; p<0.001).

**Conclusions:**

We found high variations in hospital ED performance in regard to start antibiotic treatment. The implementation of measures to reduce treatment times has the potential to improve patient care.

## Introduction

Overcrowding of emergency departments (ED) has become a worldwide challenge [1]. International measures of ED crowding have demonstrated a steady increase of ED visits over the past few decades [2]. In the United States (US) e.g., there has been an enormous increase in ED visits, coinciding with a downsizing in the number of ED institutions and consecutively longer ED lengths of stay (LOS). This was called a major problem (“national epidemic”) by the Institute of Medicine in 2006 [3]. Previously descripted reasons for crowding ED are multifaceted, e.g. involving non-urgent visits, “frequent-flyer” patients, influenza season, inadequate staffing, inpatient boarding, and hospital bed shortages [4]. Over the past 20 years, patients arriving in the ED have faced increasingly long average wait times, resulting in extended ED visit lengths [5–7]. Most important, these increases have been most pronounced for patients with severe, acute, and critical illnesses such as myocardial infarction and severe infections [5, 6, 8]. Focusing on acute infections, ED crowding was described to be associated with delayed and non-receipt of antibiotics in patients admitted with community-acquired pneumonia [9, 10].

Investigating specific components and process steps, recently, a French group found significant associations between age and triage priority and the ED LOS of patients discharged from the ED [11]. Further literature has investigated the human toll of ED crowding, demonstrating relationships between crowding and negative patient-relevant outcomes, including poorer care, adverse events, medication errors and lower satisfaction [12–15].

The main purpose of this study was to perform a prospective international analysis of factors being associated with delayed administration of antibiotic therapy and ED care in patients with an acute infection requiring appropriate (e.g. antibiotic) therapy.

## Methods

### Study design and setting

We prospectively performed an observational cohort study of unselected adult medical admissions through the ED of three tertiary care hospitals in Switzerland, France, and US between March and October 2013. The Swiss hospital (Kantonsspital Aarau) is a 600-bed tertiary care hospital with most medical admissions entering the hospital over the ED. The French hospital (Hopital Pitié-Salpêtrière, Paris) is a large inner-city 1800-bed referral academic centre. The US hospital (Morton Plant Hospital, Clearwater, Florida) is a 687-bed community referral centre. As an observational quality control study, the Institutional Review Boards (IRB) of the three hospitals approved the study and waived the need for individual informed consent (main Swiss IRB: Ethikkommission Kanton Aargau (EK 2012/059); French IRB: CCTIRS—Le Comité consultatif sur le traitement de l'information en matière de recherche (C.C.T.I.R.S.) (CPP ID RCB: 2013-A00129-36); US IRB MPM-SAH Institutional Review Board, Clearwater Florida [IRB number 2013_005]). The study was registered at the ClinicalTrials.gov registration website (http://www.clinicaltrials.gov/ct2/show/NCT01768494) and the study protocol has been published previously [16] as well as the main analysis [17].

### Data collection and processing

We included all consecutive medical ED patients with a history of an acute infection. Patients presenting to the surgical ward and patients <18 years of age were excluded. All procedures were carried out as part of standard patient care. In all patients, we recorded type of infection, collected clinical parameters, pertinent initial vital signs, and laboratory values. Urinary tract infections comprised cystitis, pyelonephritis, prostatitis. Gastrointestinal tract infections mostly comprised gastroenteritis, biliary tract infections, and pancreatitis. Erysipelas displayed the largest part of skin infections, followed by cellulitis. Socio-demographic data were available using routinely gathered information from the hospital electronic medical system used for coding of diagnosis-related groups (DRG) codes. ED nurses were involved making an electronically note of ED care timeliness that included waiting time (time of arrival to time seen by a health care professional), time to drug (time of arrival to time of first drug application) and length of ED stay (LOS, time of arrival to time leaving the ED).

### Statistical analysis

We used descriptive statistics including mean with standard deviation, median with interquartile range (IQR) and frequencies on each ED measure. Patients were divided in three groups depending on national setting. To identify independent relationship between hospital- and patient characteristics and ED timeliness univariate regression models were used to assess possible predictors that were further analysed in multivariable models (adjusted for type of infection, age, gender, vital signs, laboratory values [infection/inflammation, renal, electrolytes], comorbidities) using 95% confidence intervals (CIs). In case of a dichotomous factor (e.g. no tumor, tumor), the absence of the factor was chosen as reference. In case of a multi-level factor (e.g. infections), the most common one (e.g. pneumonia) was set as reference. Tests were carried out at 5% significance levels. Analyses were performed with STATA 12.1 (Stata Corp., College Station, TX, USA).

## Results

### Population

A total of 544 medical ED patients with a history of an acute infection were included (median age 66 years, 49.3% male gender). The main type of infection at ED admission was pneumonia (40.1%), followed by urinary tract (UTI) (25.9%) and gastrointestinal infections (10.7%). Hypertension (34.6%), diabetes (16.7%), and renal failure (15.8%) were the main comorbidities. 47.2% of patients had two or more criteria for a systematic inflammatory response syndrome (SIRS) and tended to have elevated inflammatory biomarker. A detailed country specific baseline information is visualised in Table 1.

**Table 1:** Baseline characteristics.

Parameter	International (n = 544)	CH (n = 213)	F (n = 195)	US (n = 136)	p-value
**Demographics**					
Age, median (IQR), yr	66.0 (50.0–79.0)	69.0 (56.0–78.0)	57.0 (38.0–68.0)	76.0 (58.0–84.0)	<0.001
Male, No (%)	268 (49.3)	125 (58.7)	95 (48.7)	48 (35.3)	<0.001
**Type of infection, No (%)**					
Pneumonia	218 (40.1)	120 (56.3)	49 (25.1)	49 (36.0)	<0.001
Urinary tract infection	141 (25.9)	9 (4.2)	84 (43.1)	48 (35.3)	
Gastrointestinal tract infection	58 (10.7)	29 (13.6)	22 (11.3)	7 (5.1)	
Skin infection	53 (9.7)	32 (15.0)	17 (8.7)	4 (2.9)	
Others	74 (13.6)	23 (10.8)	23 (11.8)	28 (20.6)	
**Comorbidities, No (%)**					
Gastrointestinal disease	97 (17.8)	59 (27.7)	25 (12.8)	13 (9.6)	<0.001
Coronary artery disease	29 (5.3)	16 (7.5)	12 (6.2)	1 (0.7)	0.019
Congestive heart failure	36 (6.6)	20 (9.4)	5 (2.6)	11 (8.1)	0.016
Hypertension	188 (34.6)	100 (46.9)	41 (21.0)	47 (34.6)	<0.001
Chronic obstructive pulmonary disease	47 (8.6)	27 (12.7)	17 (8.7)	3 (2.2)	0.003
Diabetes mellitus	91 (16.7)	38 (17.8)	26 (13.3)	27 (19.9)	0.25
Renal failure	86 (15.8)	58 (27.2)	13 (6.7)	15 (11.0)	<0.001
Tumor	45 (8.3)	17 (8.0)	21 (10.8)	7 (5.1)	0.180
**Clinical findings**					
Heart rate, median (IQR), beats/min	92 (78–107)	91 (78–105)	95 (78–109)	91 (78–111)	0.440
Body temperature, median (IQR), °C	37.3 (36.7–38.1)	37.8 (37.1–38.6)	37.2 (36.7–37.9)	36.8 (36.3–37.3)	<0.001
Oxygen saturation, median (IQR), %	96 (93–98)	94 (91–97)	98 (95–99)	96 (93–98)	<0.001
Systolic blood pressure, median (IQR), mmHg	130 (115–148)	133 (118–148)	127 (115–144)	131 (112–155)	0.180
Diastolic blood pressure, median (IQR), mmHg	76 (65–87)	76 (67.5–87)	76 (67–87)	74 (62–85)	0.150
SIRS: 0/1 criteria, No (%)	287 (52.8)	92 (43.2)	109 (55.9)	86 (63.2)	<0.001
SIRS: 2–4 criteria, No (%)	257 (47.2)	121 (56.8)	86 (44.1)	50 (36.8)	
**Initial laboratory findings, median (IQR)**					
White blood cells count, cells x 10^9^/L	10.9 (7.5–14.5)	11.2 (8.1–14.5)	10.9 (7.1–14.7)	10.1 (7.5–14.1)	0.470
C-reactive protein, mg/dL	110 (30–231)	103 (33.8–171)	21 (3–85)	108.5 (26.5–238)	<0.001
Sodium, mmol/L	137 (134–139)	137 (134–139)	137 (135–139)	138 (135.5–140)	0.019
Glucose, mmol/L	6.5 (5.6–8.2)	6.5 (5.8–8.2)	6.2 (5.6–8)	4.8 (4.3–5.3)	0.570
Creatinine, μmol/L	86 (67–116)	99 (79–140)	69 (55–93)	88 (71–133)	<0.001

CH, Switzerland; F, France; US, United States of America; IQR, interquartile range; SIRS, Systemic Inflammatory Response Syndrome.

### Time to start medication

In general, median time to antibiotic administration was 214 minutes (95% CI: 199, 228), with a maximum of 307 minutes; 95% CI: 263, 328) in France, 230 minutes (95% CI: 210, 264) in Switzerland, and 161 minutes (95% CI: 144, 170) in the US. When looking at gastrointestinal infections in the overall cohort (international), median time to drug (326 minutes [95% CI: 284, 372]) was significantly (p<0.001 [ANOVA]) increased compared to more localised infections such as pneumonia (209 minutes [95% CI: 192, 228]), UTI (204 minutes [95% CI: 169, 233]), and skin infections (207 minutes [95% CI: 168, 253]) (Table 2). We also found significant differences in time to discharge when comparing different infection sites in the overall patient cohort (p = 0.001 [ANOVA]). This was mainly due to patients from the Swiss centre. No differences were observed in time to first physician contact among different infections. Consistently, in all subgroups of acute infections, we found highly significant time differences in starting antibiotics between European and US hospitals.

**Table 2:** Timeliness of ED care.

		International (n = 544)	CH (n = 213)	F (n = 195)	US (n = 136)	
Diagnosis	Time (minutes) from hospital admission to [median (95% CI); min, max]					p-value
**Overall infections (n = 531)**	First physician contact	55 (49, 57); 1, 350	62 (59, 69); 6, 350	67 (55, 77); 4, 195	19 (16, 22); 1, 165	<0.001
	Medication (antibiotics)	214 (199, 228); 26, 476	230 (210, 264); 69, 476	307 (263, 328); 51, 474	161 (144, 170); 26, 425	<0.001
	ED discharge	322 (308, 335); 69, 718	361 (346, 382); 150, 718	319 (300, 340); 69, 653	260 (245, 274); 124, 638	<0.001
**Pneumonia (n = 218)**	First physician contact	53 (44, 58); 1, 350	61 (57, 67); 6, 350	58 (42, 82); 4, 177	21 (14, 23); 1, 136	<0.001
	Medication (antibiotics)	209 (192, 228); 26, 474	220 (200, 252); 77, 465	310 (220, 359); 51, 474	141 (124, 162); 26, 371	<0.001
	ED discharge	326 (303, 344); 69, 698	350 (331, 367); 167, 698	326 (268, 359); 69, 580	249 (221, 276); 124, 497	<0.001
**UTI (n = 141)**	First physician contact	49 (39, 60); 1, 329	57 (12, 182); 12, 329	78 (63, 88); 13, 195	18 (15, 24); 1, 125	<0.001
	Medication (antibiotics)	204 (169, 233); 69, 447	246 (142, 345); 97, 377	314 (221, 339); 69, 447	165 (145, 182); 88, 394	<0.001
	ED discharge	294 (274, 318); 75, 639	434 (273, 527); 254, 572	311 (292, 340); 75, 639	247 (224, 268); 143, 523	0.001
**GIT (n = 58)**	First physician contact	60 (51, 67); 8, 345	64 (57, 82); 25, 345	52 (34, 70); 17, 130	14 (9, 119); 8, 127	0.032
	Medication (antibiotics)	326 (284, 372); 93, 476	330 (295, 374); 93, 476	369 (311, 396); 311, 396	228 (218, 248); 218, 248	0.213
	ED discharge	389 (354, 440); 133, 718	441 (383, 478); 244, 718	374 (299, 440); 133, 653	318 (175, 398); 135, 410	0.027
**Skin infections (n = 53)**	First physician contact	60 (54, 83); 3, 304	74 (57, 114); 13, 304	54 (47, 86); 24, 123	35 (3, 55); 3, 55	0.040
	Medication (antibiotics)	207 (168, 253); 69, 468	189 (159, 245); 69, 414	242 (144, 463); 109, 468	257 (250, 264); 250, 264	0.231
	ED discharge	327 (287, 362); 135, 590	325 (278, 365); 150, 581	357 (279, 424); 135, 590	267 (189, 359); 189, 359	0.146
**Other infections (n = 74)**	First physician contact	46 (28, 67); 3, 299	71 (55, 110); 8, 299	70 (32, 88); 9, 153	19 (16, 37); 3, 165	0.002
	Medication (antibiotics)	187 (169, 238); 39, 473	234 (181, 334); 90, 473	248 (70, 371); 69, 380	165 (129, 199); 39, 425	0.077
	ED discharge	317 (278, 362); 86, 711	412 (368, 530); 177, 711	282 (213, 368); 86, 613	284 (258, 329); 186, 638	0.012

CH, Switzerland; F, France; US, United States of America; CI, confidence interval; UTI, urinary tract infection; GIT, gastrointestinal tract infection; ED, emergency department.

### Timeliness of ED care

**Fig 1:**
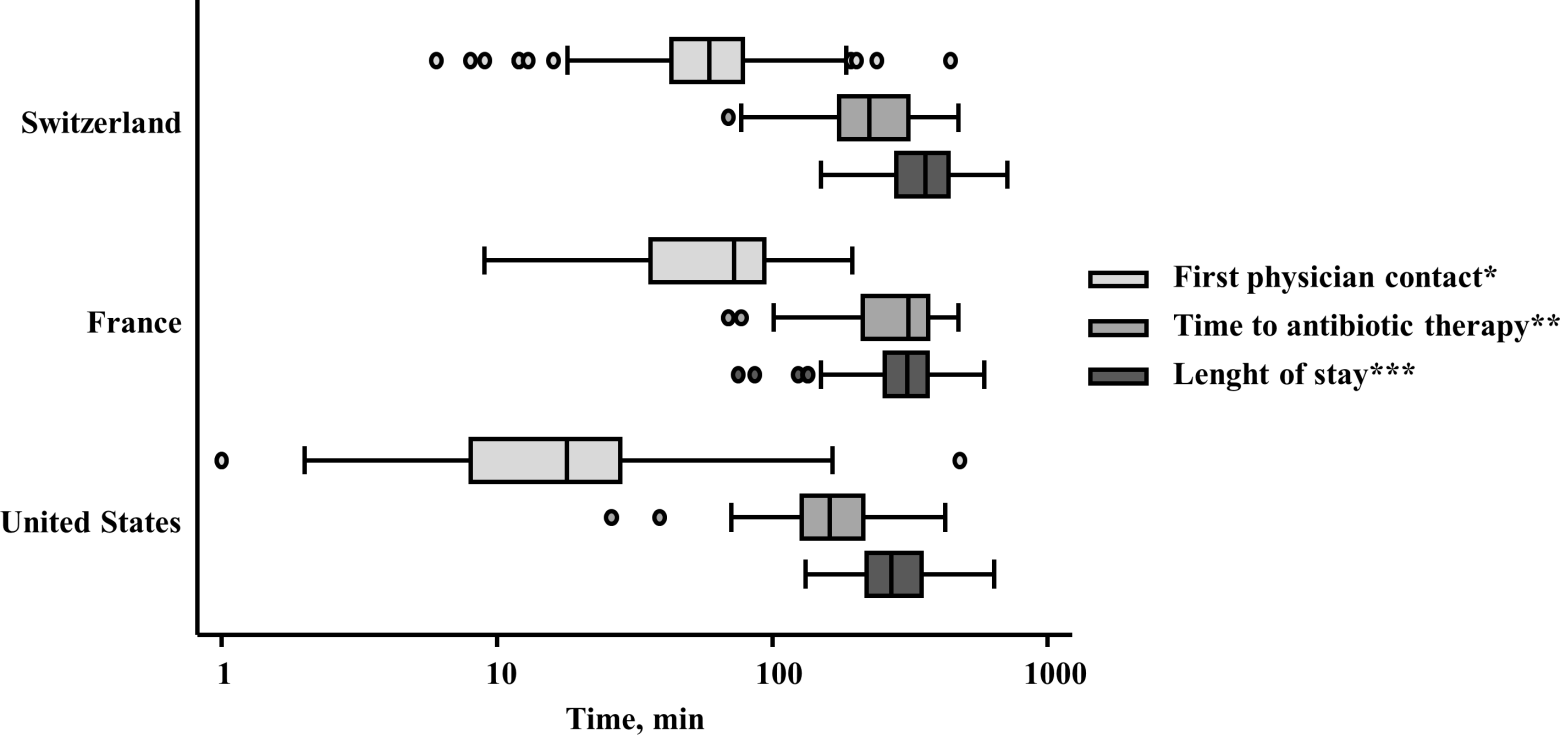
Distribution of ED measures of timely care in patients with acute infections across different countries. The bottom and top of the box represent the 25th and 75th percentiles of the hospital-reported mean times for that measure, with the middle line representing the median. * Time of arrival to time seen by a doctor. ** Time of arrival to time being given medication. *** Time of arrival to time leaving the emergency department for home or for an in-hospital bed.

As shown in Fig 1 and Table 2, the overall median waiting time to the first physician contact was 55 minutes (95% CI: 49, 57), notably, there were large differences between the two European hospitals (62 and 67 minutes in Switzerland and France, respectively) and the US hospital (19 minutes; 95% CI: 16, 22). Analog to above mentioned differences in time to start antibiotic therapy, this trend was also obvious investigating subgroups of infections. Finally, international median ED LOS was 322 minutes (95% CI: 308, 335), with a maximum in Switzerland (361 minutes; 95% CI: 346, 382), followed by France (319 minutes; 95% CI: 300, 340), and the US with 260 minutes (95% CI: 245, 274). Corresponding to median time to antibiotics, median ED LOS was larger in gastrointestinal infections than in other infection subgroups. Notable, in French patients with UTI, median time to antibiotic therapy was longer than median ED LOS, resulting from delayed drug administration in clinically stable patients only after ED discharge to the medical ward. All results in detail are shown in Table 2.

### Predictors for delayed antibiotic drug administration

As shown in Table 3, using multivariable adjusted regression analyses, existence of a gastrointestinal tract infection was a significant predictive parameter for delayed start of therapy compared to patients with pneumonia, whereas patients with more localised skin infections, such as erysipelas, tended to results in faster drug administration (e.g. in Switzerland). Focusing demographic characteristics, Swiss patients with an age between 66 and 79 years tended to be at risk for a later onset of adequate therapy using univariate analyses. However, older US patients were treated earlier than younger ones. In multivariable regression models these effects could not be substantiated. Additionally, Swiss patients with a larger inflammation (more positive SIRS criteria) or a chronic obstructive pulmonary disease (COPD) showed a decreased time to first drug administration. In France, diabetic patients and patients with a hyperglycaemia had to wait longer for an appropriate anti-infective therapy compared to non-diabetic ones. Finally, patients entering the ED during “rush hours” (6–12 pm) tended to wait longer for an appropriate medication.

**Table 3:** Predictors of delayed antibiotic drug administration.

Predictor	International (n = 544)		CH (n = 213)		F (n = 195)	
	Coefficient (95% CI)	p-value	Coefficient (95% CI)	p-value	Coefficient (95% CI)	p-value
**Pneumonia**	reference	-	reference	-	reference	-
**UTI**	40.7 (-13.0–94.4)	0.137	7.0 (-89.2–103.2)	0.886	163.6 (-163.3–490.4)	0.308
**GIT**	119.5 (58.0–181.0)	<0.001	92.6 (34.4–150.9)	0.002	267.3 (-151.5–686.0)	0.197
**Skin infections**	-46.5 (-101.0–8.0)	0.094	-65.5 (-118.9–12.1)	0.016	35.6 (-311.1–382.2)	0.832
**Other infections**	31.2 (-33.3–95.8)	0.341	1.1 (-62.0–64.3)	0.972	82.9 (-311.7–477.5)	0.665
**Age ≤50y**	reference	-	reference	-	reference	-
**Age ≤66y**	10.4 (-47.5–68.4)	0.724	35.8 (-25.1–96.6)	0.248	-295.1 (-487.4–102.8)	0.005
**Age ≤79y**	23.3 (-33.9–80.5)	0.423	30.7 (-29.8–91.2)	0.318	-87.1 (-294.1–119.8)	0.389
**Age >79y**	-36.6 (-103.9–30.6)	0.284	-28.5 (-99.3–42.3)	0.428	-120.3 (-403.7–163.1)	0.385
**Female**	reference	-	reference	-	reference	-
**Male**	-12.8 (-53.7–28.1)	0.538	-15.8 (-58.0–26.4)	0.461	43.1 (-130.6–216.7)	0.610
**Day time 0–6 am**	reference	-	reference	-	reference	-
**Day time 6–12 am**	26.6 (-55.8–109.0)	0.525	4.9 (-73.7–83.4)	0.903	-17.8 (-162.1–126.4)	0.798
**Day time 0–6 pm**	22.2 (-59.0–103.5)	0.59	21.9 (-53.8–97.6)	0.568	NA	NA
**Day time 6–12 pm**	39.5 (-44.2–123.2)	0.354	38.8 (-37.6–115.1)	0.318	NA	NA
**0 SIRS criteria**	reference	-	reference	-	reference	-
**1 SIRS criteria**	-35.8 (-94.5–23.3)	0.235	-38.0 (-96.0–19.9)	0.197	-49.9 (-290.8–191.0)	0.669
**2 SIRS criteria**	-24.0 (-82.7–34.6)	0.42	-26.8 (-87.1–33.5)	0.382	64.2 (-206.7–335.1)	0.626
**3/4 SIRS criteria**	-59.1 (-120.9–2.6)	0.06	-60.7 (-123.3–2.0)	0.058	98.4 (-195.3–392.1)	0.492
**≤130mmHg systolic**	reference	-	reference	-	reference	-
**>130mmHg systolic**	11.1 (-32.1–54.2)	0.614	9.9 (-34.0–53.8)	0.656	-164.3 (-391.3–62.4)	0.146
**≤76mmHg diastolic**	reference	-	reference	-	reference	-
**>76mmHg diastolic**	-5.7 (-49.7–38.3)	0.799	9.4 (-34.1–52.9)	0.669	57.0 (-121.9–236.0)	0.513
**Sodium (136–146mmol/L)**	reference	-	reference	-	reference	-
**Sodium (<136, >146mmol/L)**	-21.9 (-61.9–18.1)	0.281	-20.4 (-60.3–19.5)	0.315	-23.8 (-205.3–157.6)	0.786
**Glucose (4–7mmol/L)**	reference	-	reference	-	reference	-
**Glucose (<4, >7mmol/L)**	-26.6 (-67.9–14.6)	0.205	-17.6 (-60.8–25.5)	0.421	203.0 (-2.1–408.2)	0.052
**Creatinine (≤86mg/L)**	reference	-	reference	-	reference	-
**Creatinine (>86mg/L)**	21.4 (-24.3–67.2)	0.357	6.8 (-40.1–53.6)	0.776	43.7 (-130.0–217.4)	0.604
**CRP (≤69mg/L)**	reference	-	reference	-	reference	-
**CRP (>69mg/L)**	13.3 (-25.7–52.3)	0.502	1.6 (-37.9–41.2)	0.935	58.9 (-98.6–216.4)	0.443
**No obesity**	reference	-	reference	-	reference	-
**Obesity**	-11.2 (-70.0–47.5)	0.707	0.1 (-54.1–54.3)	0.998	NA	NA
**No CAD**	reference	-	reference	-	reference	-
**CAD**	-13.6 (-83.4–56.1)	0.701	11.7 (-57.0–80.3)	0.738	-137.7 (-476.1–200.7)	0.405
**No CHF**	reference	-	reference	-	reference	-
**CHF**	27.7 (-39.3–94.6)	0.417	22.0 (-42.6–86.6)	0.502	239.6 (-255.5–734.6)	0.324
**No COPD**	reference	-	reference	-	reference	-
**COPD**	-44.7 (-101.3–11.8)	0.12	-66.2 (-120.2–12.3)	0.016	-51.1 (-421.1–319.0)	0.776
**No tumor**	reference	-	reference	-	reference	-
**Tumor**	27.6 (-35.1–90.2)	0.387	40.4 (-26.1–106.9)	0.232	-75.0 (-339.2–189.1)	0.559
**No diabetes**	reference	-	reference	-	reference	-
**Diabetes**	27.8 (-23.5–79.1)	0.287	-9.1 (-64.6–46.5)	0.747	222.3 (-0.5–445.0)	0.050
**No GIT disease**	reference	-	reference	-	reference	-
GIT disease*	-1.6 (-46.3–43.0)	0.942	-8.0 (-50.4–34.3)	0.709	252.0 (-36.1–540.0)	0.083
**No hypertension**	reference	-	reference	-	reference	-
**Hypertension**	29.4 (-10.4–69.3)	0.146	38.0 (-1.8–77.9)	0.061	19.3 (-168.0–206.6)	0.831
**No renal failure**	reference	-	reference	-	reference	-
**Renal failure**	-30.3 (-79.0–18.3)	0.22	-12.6 (-61.3–36.1)	0.609	-59.6 (-297.9–178.7)	0.607

CH, Switzerland; F, France; US, United States of America; CI, confidence interval; UTI, urinary tract infection; GIT, gastrointestinal tract; SIRS, systemic inflammatory response syndrome; CRP, C-reactive protein; CAD, coronary artery disease; CHF, congestive heart failure; COPD, chronic obstructive pulmonary disease; NA, not applicable; US data not applicable due to small patient number.

## Discussion

In this international prospective observational cohort study of ED patients with a history of an acute infection we compared predictors for delay in initial care. In a second step, timeliness of ED care was investigated. This study was conducted in three tertiary hospitals with a different ED management (e.g. triage system), health care system, and socio-demographic spectrum (e.g. culture, race).

In general, we found a median ED LOS of about 5.5 hours that is higher compared to previously published data in France and the US [11, 18, 19], in Switzerland there exists no comparable data. The main reason for these observation is that most patients with acute infections have a diffuse and non-specific symptomatic (e.g. fever, shivering, fatigue) and therefore require supplementary time for diagnostic- and therapeutic steps. Most previous publications investigated unselected ED patients independent of discipline, mirroring a fuzzy image of patient subgroups like acute infectious diseases [7, 20, 21].

Waiting time, time to drug, and ED LOS were shorter in the US compared to the European centres. In addition to a slightly lower acuity and severity of admitted US patients (lower body temperature, less SIRS criteria), and significant differences in socio-demographics of ED patients among all centres (US population was older and more females), implementation of clinical pathways and fast-track admission in the US could be a possible explanation for a superior ED timeliness. Yet, baseline crowding status of the participating hospitals was not available and we can thus not exclude bias in this regard. Such information should be included in future larger trials. Another explanation may relate to the high administrative work associated with ED admission in Switzerland [22], resulting in a prolonged time from therapy to ED discharge.

Mainly, the presence of a gastrointestinal tract infection was a significant predictor of a delayed initiation of an adequate therapy, likely because abdominal site infection may be diagnostically more obscure and symptoms masked or atypical. In contrast, more localised infections (e.g. skin infections)–requiring less extended supplementary diagnostics–tended to be treated faster, also resulting in a decreased ED LOS. Severe cases (more positive SIRS criteria, relevant comorbidities such as COPD) were treated significantly faster in the Swiss population. In France, diabetic patients showed longer waiting times, potentially mirroring a more polymorbid (e.g. metabolic syndrome) patient population. Due to small the small number of patients included from the US site, we focused on the comparison of the two European centres.

As a second point, analogous to previous literature [20, 23, 24], length of stay was increased in Swiss elderly patients, suggesting a higher complexity due to comorbidities and a more prolonged decision-making process than in younger ones. Finally, ED patients presenting between 6:00 and 12:00 p.m. tended to have a longer ED stay, correlating with increased patient admissions [25].

Our study has some important limitations. First, this was an observational study that was focusing on the question if an improved initial triage of patients at the earliest stage of ED admission with incorporation of a triage system, initial clinical parameters, vital signs, and prognostic blood markers will improve patient triage [16, 26]. Second, we investigated a small patient sample, with partially incomplete available data, limiting subgroup analyses`conclusions. Third, we did not take into account the number of medical and non-medical staff, available in these EDs to take care of the patients. Last, we did not consider in-hospital bed capacity that might have had decelerating effects on transfer from the ED to the medical ward. All these limitations could have implications in interpreting our results and may limit external validity. However, we performed an international multicentre study with many available ED parameters, and updated reasons for ED consultation, displaying a major strength of this study.

To further decrease time to first physician contact and time to antibiotics, especially in urgent patients at risk for worse outcome, fast and high sensitive point-of-care testing (POCT) devices measuring prognostic biomarkers could be a promising tool [27]. Herein, a new prognostic biomarker (proadrenomedullin, ProADM) [28] is supposed to support differentiating urgent from non-urgent patients showing non-conclusive clinical symptoms and to improve existing triage systems. Alternatively, as previously published by Chartier et al., ED flow can be significantly improved by re-purposing a fraction of existing staff, resources, and infrastructure for patients with lower acuity presentations [29]. From the initial triages perspective, a recent study that implemented a computer-assisted triage system using acute physiology, pre-existing illness and mobility showed a measurable impact on cost of care for patients with very low risk of death. Patients were safely discharged earlier to their own home and the intervention was cost-effective [30]. Finally, another study investigated the effect of implementation of a triage/treatment pathway in adult patients with cancer and febrile neutropenia improving initial ED triage. In this study, antibiotic delays were reduced and quality of care for patients was improved [31]. To definitively proof effectiveness and safety of all these promising tools, well-powered large randomized controlled trials are inevitable.

### Conclusion

Time to adequate drug administration and ED LOS were principally associated with the clinical picture and the need for diagnostics. In Switzerland, older patients were treated later, suggesting a higher complexity–rudimentary seen in French diabetic patients—with a longer clinical decision-making process than in younger patients. Even, if necessary to optimise evaluation and reach the best final disposition decision, can lengthen the overall time spent in the ED.

This international study provides new insights into the relative effect of diffuse clinical pictures, comorbidities, and demographics on international ED timeliness. Our results suggest that new strategies to reduce time to antibiotic medication and ED LOS–especially in patients with an acute infection—should include suitable advices (e.g. POCT) for ancillary biomarker measurement.

## Abbreviations

CI: confidence interval
COPD: chronic obstructive pulmonary disease
DRG: diagnosis-related groups
ED: emergency department
IQR: interquartile range
LOS: length of stay
POCT: point-of-care testing
ProADM: proadrenomedullin
SIRS: systematic inflammatory response syndrome
US: United States
UTI: urinary tract infection
